# The costs and benefits of environmental sustainability

**DOI:** 10.1007/s11625-021-00910-5

**Published:** 2021-03-16

**Authors:** Paul Ekins, Dimitri Zenghelis

**Affiliations:** 1 UCL Institute of Sustainable Resources, University College London, London, UK; 2 London School of Economics and Political Science, London, UK

**Keywords:** GEO-6, Low-carbon transition, Path dependency and Lock in, Dynamic costs and benefits, Endogenous growth

## Abstract

The natural science in GEO-6 makes clear that a range and variety of unwelcome outcomes for humanity, with potentially very significant impacts for human health, become increasingly likely if societies maintain their current development paths. This paper assesses what is known about the likely economic implications of either current trends or the transformation to a low-carbon and resource-efficient economy in the years to 2050 for which GEO-6 calls. A key conclusion is that no conventional cost–benefit analysis for either scenario is possible. This is because the final cost of meeting various decarbonisation and resource-management pathways depends on decisions made today in changing behaviour and generating innovation. The inadequacies of conventional modelling approaches generally lead to understating the risks from unmitigated climate change and overstating the costs of a low-carbon transition, by missing out the cumulative gains from path-dependent innovation. This leads to a flawed conclusion as to how to respond to the climate emergency, namely that significant reductions in emissions are prohibitively expensive and, therefore, to be avoided until new, cost-effective technologies are developed. We argue that this is inconsistent with the evidence and counterproductive in serving to delay decarbonisation efforts, thereby increasing its costs. Understanding the processes which drive innovation, change social norms and avoid locking in to carbon- and resource-intensive technologies, infrastructure and behaviours, will help decision makers as they ponder how to respond to the increasingly stark warnings of natural scientists about the deteriorating condition of the natural environment.

## Introduction

The sixth UN Global Environment Outlook (GEO-6) (UNEP 2019) focused on the close relationship between human and environmental health, presenting much evidence that a healthy planet is necessary for healthy people and that, conversely, an unhealthy planet damages human health. This paper echoes, and indeed adds to, the findings of GEO-6 by setting out the benefits to early policy action, not only to limit potentially catastrophic environmental risks, but also to shape new markets, create business opportunities and induce new technologies and behaviours which will benefit society beyond their environmental value. Section “The costs of ‘grow now, clean up later’” of the paper reviews the evidence from GEO-6 and elsewhere of the costs of environmental damage resulting from a still largely dominant global development model of ‘grow now, clean up later’. This describes the approach through which societies’ efforts to achieve economic growth damage natural systems and their environmental functions, reducing their ability to deliver multiple benefits in terms of ecosystem goods and services, entailing costs to health, with knock-on effects on human societies and economies. These costs could be much amplified if global warming induces climate ‘tipping points’, feedback effects through which warming itself is amplified. GEO-6 did not investigate this in detail, but evidence is now beginning to emerge as to the extra costs that these tipping points could entail.

The costs of environmental damage become the benefits of environmental protection and restoration, if they are thereby mitigated or avoided. There are three broad environmental strategies to deliver these benefits, the ‘triple-de’: *decarbonisation*, to reduce the level of global warming; *detoxification*, to reduce the emissions or impacts of other pollutants; and *dematerialisation*, to reduce the environmental impacts associated with resource extraction, conversion and processing. Section “The costs of environmental protection” of the paper explores the issue as to what the implementation of ‘triple-de’ strategies would cost with a focus on attempts to model the costs of decarbonisation. This is seen to hinge on the role of innovation—in technologies, behaviours and institutions—that makes the costs endogenous to the policy approach taken; the costs associated with the transition to a sustainable economy will be a function of today’s decisions. The paper also considers the costs of locking in to environmentally damaging technologies, behaviours and institutions that then have to be abandoned or retrofitted—costs that are avoided if economic development and clean environmental performance are managed together working with the investment cycle. The final section concludes and draws recommendations for decision-makers.

## The costs of ‘grow now, clean up later’

The common policy approach to economic development has been to concentrate on getting rich first, and hope to have the resources to fix the environment later—the ‘grow now, clean up later’ mind set. This is the way the old industrial countries did it, and the standard assumption, especially in developing and emerging economies, and despite increasing rhetoric espousing ‘sustainable development’ and the Paris Agreement, is that there is no better way to develop economically.

Notwithstanding the benefits that economic growth has brought millions of people in both old industrial and emerging economies—lifting them out of poverty, reducing infant mortality and other preventable deaths, increasing life expectancy, literacy, access to water and sanitation, eradication of diseases—evidence in respect of the environment and natural resources now suggests that this approach has brought human societies to the brink of catastrophe, putting at serious risk all these benefits and, indeed, the continuance of human civilisation itself. This section will explore these risks, and the current costs that accompany them. It will reveal the new priorities of human development to be detoxification, decarbonisation and dematerialisation.

The irony is that the assumption that countries at an early stage of development need to suffer gross pollution to get richer flies in the face of more than two decades of evidence, as will be seen.

### The costs of ‘clean up later*’*

#### Early evidence

One of the earliest papers to investigate the assumption that pollution was a necessary accompaniment to early growth was O’Connor (1996), who tested it in respect of the newly industrialising or recently industrialised economies of East and South East Asia. He considered the environmental dimension of the growth process of Japan, what were then called ‘the four dragons’—Hong Kong, Korea, Taiwan, and Singapore—and some later industrialising countries in the region (Indonesia, Malaysia, and Thailand). He reviewed evidence on relative pollution intensity and energy intensity, estimates of the environmental damage costs incurred by these East Asian economies and how these related to actual measured output, and evidence on environmental expenditures in these countries, both actual and projected. His conclusions were startling. He found that all the countries studied had to some extent taken a ‘grow now, clean up later’ approach, but some markedly more so than others.

What was clear was that the countries with the more pollution-intensive growth patterns later faced significant challenges that could constrain future growth through “rapidly escalating external costs from accumulated pollution damage and/or rapidly escalating investments in remediation of that damage” (p.15). Moreover, there was no evidence at all that the countries which had invested early in pollution control along with their industrialisation (e.g. Hong Kong and Singapore) had suffered lower growth rates than those countries that had invested much less. The oft-hypothesised growth-environment trade-off at early stages of development was simply not apparent in the data.

O’Connor 1996 (pp.31–32) identifies a number of reasons why it may be more expensive to address pollution problems after they have been created rather than preventing them from occurring. Most obviously, collecting and treating waste is likely to be cheaper before it is widely dispersed in the natural environment. But there are also issues related to abatement costs. Cleaning up polluting plant usually involves either scrapping equipment before its due date, or retrofitting it, or fitting end-of-pipe treatment technology. It will often be the case that initial investment in cleaner, if initially more expensive, technology that avoids these extra costs, ‘leap-frogging’ the phase of gross pollution, can be economically preferable.

Moreover, the ‘lock-in’ from past investments, especially in infrastructure, may mean that environmental improvement can only gradually be achieved leaving a long period of high environmental costs that could have been avoided with different initial infrastructure decisions. O’Connor (1996, p.36) also cites evidence that much environmental improvement can be achieved through measures with very low payback times that make economic sense in their own right. All this leads him to conclude that any negative effects from increased environmental expenditures “should be largely if not wholly offset by the positive effect on the productivity of public and private sector investments, through the mitigation of health and other pollution damages” (p.34). Unsurprisingly, he finds no evidence of slower economic growth in those Asian countries (e.g. Hong Kong, Singapore) that have considered environmental as well as economic performance compared with those that have not.

#### Evidence since 2010

Unfortunately, despite the evidence cited above, this lesson is far from learned. A book-length study by the World Bank of India’s environmental and economic development record (Mani 2014) asserts, (p.4): “The “grow now, clean up later” doctrine, though much debated, is now widely discredited by the experiences of many developing countries.” But much of the subsequent text goes to show that the ‘doctrine’ is still very much in evidence in the state of India’s air, water and ecosystems. The report puts the “total cost of environmental degradation in India” at 5.7% of India’s GDP (mid-point, range 2.6–8.8%), comprising costs from outdoor and indoor air pollution (29% and 21% respectively), from inadequate water supply, sanitation and hygiene (14%) and degradation of cropland (19%), pasture (11%) and forests (4%) (percentages are of the mid-point damages).

**Fig. 1:**
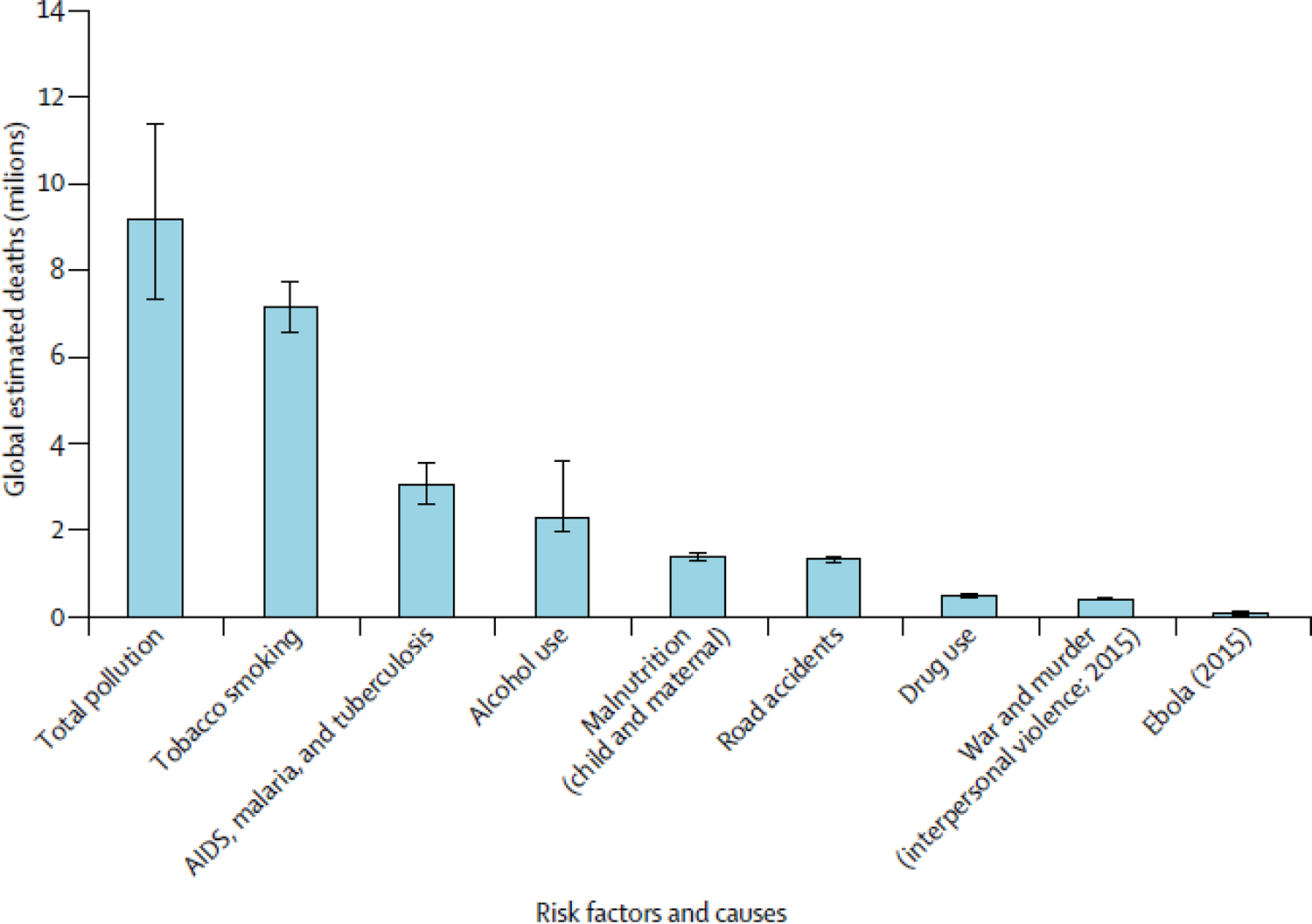
Global estimate deaths in 2015 by major risk factor and cause; Source: Landrigan et al. 2018, Fig. 5, p.473

More recently, the comprehensive assessment of the health impacts of global pollution of Landrigan et al. (2018) shows just how few lessons have been learned since the 1990s. They estimate that pollution-related disease was responsible for 16% of total global mortality, or 254 million years of life lost, in 2015. As shown in Fig. 1, this number is considerably greater than the estimated deaths from a number of other causes that have a much higher public profile. The total welfare damages of this pollution burden have been estimated for 2015 at USD4.6 trillion, equivalent to 6.2% of global GDP (Landrigan et al. 2018, p.487). Landrigan et al. (2018) echo the findings of O’Connor some 20 years earlier:

“The claim that pollution control stifles economic growth and that poor countries must pass through a phase of pollution and disease on the road to prosperity has repeatedly been proven to be untrue. … Many of the pollution control strategies that have proven cost-effective in high-income and middle-income countries can be exported and adapted by cities and countries at every level of income.” (Landrigan et al. 2018, p.463).

Detoxification, it seems, often makes economic sense quite apart from the welfare benefits of resulting in healthier people. The next section shows that the arguments for decarbonisation are just as strong.

### The ‘fat-tailed’ costs of climate change

The estimates of possible damages from climate change are so large they are difficult to comprehend. The IPCC 1.5 °C report made clear that even going beyond a temperature increase of 1.5 °C would significantly increase the risks of substantial damage from climate change, and cites estimates of the extra damage caused in 2100 by 2 °C as opposed to 1.5 °C as USD 15–38.5 trillion (2.3–3.5% of Gross World Product) (IPCC 2018, p.256).

Steffen et al. (2018) have investigated in detail the various planetary thresholds that may act as ‘tipping points’, at different levels of global warming, into a Hothouse Earth. The colours in their ‘Global map of potential tipping cascades’ illustrate the average global temperature increases at which that particular feature may tip into a different state. The arrows, based on expert elicitation, indicate how the activation of some tipping points by a relatively low level of global warming (e.g. melting of the Greenland Ice Sheet at 1–3 °C) could push the global average temperature higher, activating further tipping points such as the thermohaline circulation, thereby causing a tipping point ‘cascade’ effect.

Of course, there is very great uncertainty about the precise magnitude of these effects, and it is probably best to think of these numbers in terms of risk, in this case the risk from a probability distribution that has a ‘fat-tailed’ probability of very large costs. Weitzman emphasised the *“truly extraordinary uncertainty about the aggregate welfare impacts of catastrophic climate change, which is represented mathematically by a PDF that is spread out and heavy with probability in the tails.”* (Weitzman 2011, p.285).

To emphasise further how difficult people find it to act consistently in the face of such uncertainty, consider Table 1, from Wagner and Weitzman (2015).

**Table 1:** Probabilities of exceeding an average global temperature increase of 6 °C at different atmospheric concentrations of GHGs; Source: Wagner and Weitzman 2015, Table 3.1, p.54.

CO_2_ concn. (ppm)	400	450	500	550	600	650	**700**	750	800
Median temp. increase (°C)	1.3	1.8	2.2	2.5	2.7	3.2	**3.4**	3.7	3.9
Chance of > 6°C (%)	0.04	0.3	1.2	3	5	8	**11**	14	17

Using the IPCC’s ‘likely’ climate sensitivity, Table 1 shows the median temperature increase at different levels of atmospheric concentration of carbon dioxide equivalents (CO_2_e), but also the probability that the temperature increase will be more than 6 °C—which Wagner and Weitzman call ‘an indisputable global catastrophe’ (Wager and Weitzman 2015, p.88). This probability is seen to be 11% at an atmospheric GHG concentration of 700 ppm, which is in line with the IEA’s projection for 2100 even if governments kept their then current promises (cited in Wager and Weitzman 2015, p.55). But they also give a 0.3% chance of exceeding a 6 °C temperature increase at 450 ppm, which is roughly the *current* atmospheric GHG concentration.

To put this in perspective, it may be noted that in 2018 there were around 38 million aircraft flights per year (ICAO 2018), with one fatal accident every three million flights,^1^ a probability of 0.000033%. A 0.3% probability of a fatal accident would mean over 300 fatal accidents *each day*. How many people would fly given that kind of accident rate reported daily on the news? Yet that is the risk human societies are currently taking in respect of catastrophic climate change. Such risk taking becomes even more bizarre when it is considered that the health benefits just from reduced local air pollution of achieving the 2 °C target could be 1.4–2.5 times the cost of mitigation, the higher figure involving benefits of USD 54.1 trillion for a global expenditure of USD 22.1 trillion (UNEP 2019, Box 24.1, p.588). Deep decarbonisation makes sense whether looked at from the point of view of global insurance against catastrophe for future generations, or health benefits for those currently alive.

### The costs of resources

The ‘grow now, clean up later’ mind set has not only been cavalier in respect of pollution and indifferent to the greenhouse gas emissions causing climate change, it has also been extraordinarily wasteful in its use of resources, through a related mind set of ‘take-make-use-dispose’. The ‘grow now, clean up later’ economy has also been a linear, throw-away economy, as attested by the mountains of garbage and oceans of plastic that are now in evidence almost everywhere.

The *Global Resources Outlook* of the International Resource Panel (IRP 2019a; b) has documented the growth of resource use since 1970 and associated environmental impacts. Since 1970 global material use has more than tripled from 27 billion tonnes to 92 billion tonnes, with biomass increasing from 9.1 to 24.1 billion tonnes, fossil fuels from 6.2 to 15.0 billion tonnes, metal ores from 2.6 to 9.1 billion tonnes, and non-metallic minerals, the greatest proportionate increase due to its importance in infrastructure construction, from 9.2 to 43.8 billion tonnes (IRP 2019a, p.43–44).

**Fig. 2:**
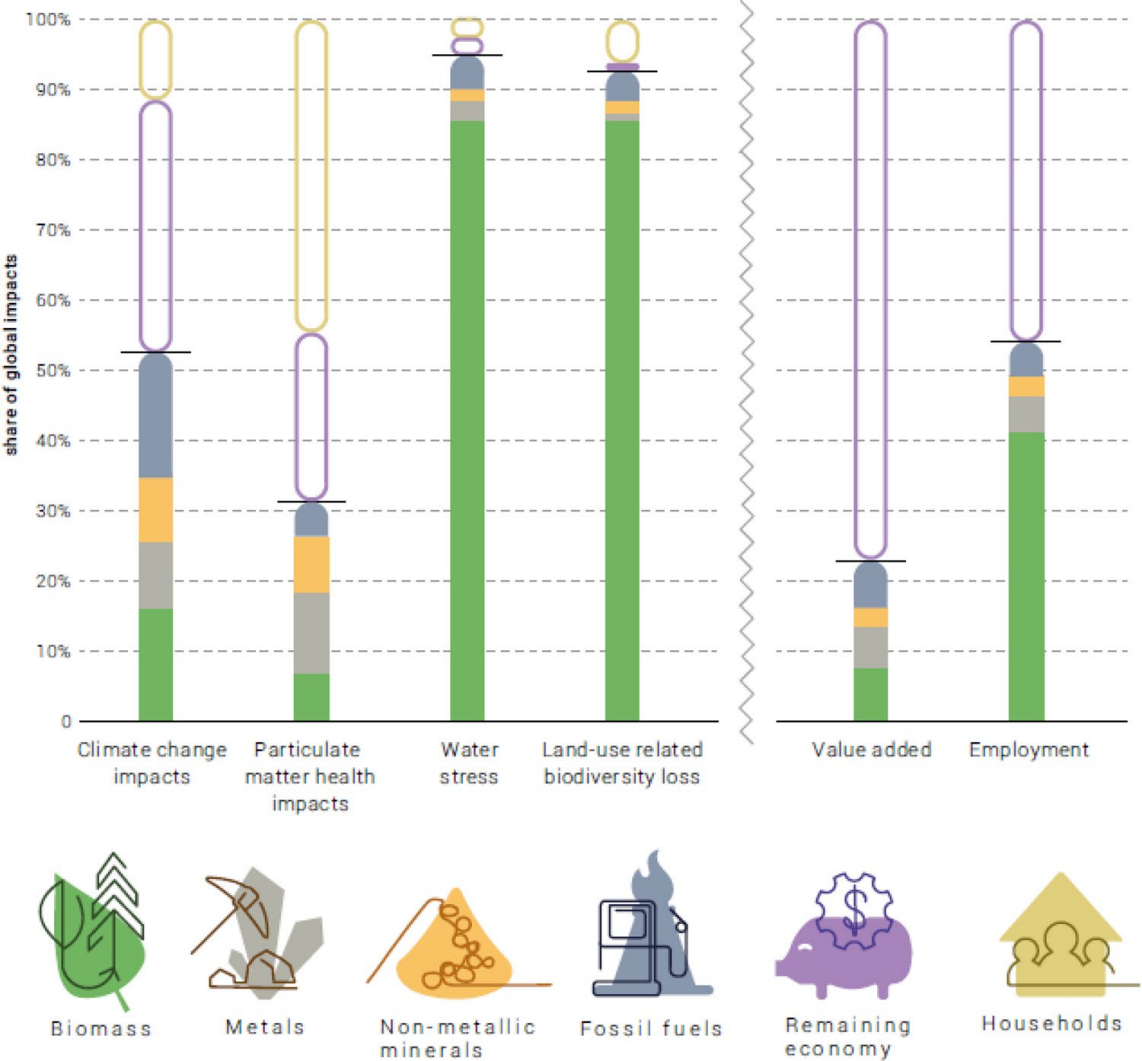
Environmental and economic impacts of resource extraction, compared with households and the rest of the economy; Source: IRP 2019b, Figure II, p.16

This extractive activity is associated with very large environmental impacts, as shown in Fig. 2. Biomass production alone (mainly agriculture) is responsible for nearly 90% of biodiversity loss and water stress. Extraction (including fossil fuels) is also responsible for 50% of greenhouse gas emissions and 30% of particulate matter health impacts. Yet these industries only contribute around 20% of global value added.

On current trends global material extraction would double again by 2060 to 190 billion tonnes, with greenhouse gas emissions increasing by 43%, and cropland and pasture land increasing by 20% and 25% respectively, with forests and other natural habitat decreasing by 10% and 20% respectively (IRP 2019a, Sect. 4.2, pp.102ff.). Many populations will experience water scarcity, with growing competition for water between cities and agriculture. It is very unlikely that the Earth’s natural systems would sustain this kind of increase in natural resource extraction and the associated environmental impacts. A liveable future will demand dematerialisation of the economy, particularly a decoupling between economic growth and resource use, and an absolute reduction in the environmental impacts of resource extraction of all kinds (how to achieve this is discussed in section “The costs of environmental protection”).

### The age of irreversibility

While the ‘grow now, clean up later’ approach was always economically unsound, more recently it has become theoretically unsound as well. At a local level, it is possible for a time to substitute natural capital for physical capital and boost prosperity, as arguably the UK did during the industrial revolution. But at a global level this is not possible. With climate change and biodiversity loss, there is no return to the *status quo ante*. There is no ‘later’. Extinction is forever, with the Intergovernmental Panel on Biodiversity and Ecosystem Services (IPBES) suggesting that:

“around 1 million species already face extinction, many within decades, unless action is taken …. Without such action there will be a further acceleration in the global rate of species extinction, which is already at least tens to hundreds of times higher than it has averaged over the past 10 million years.” (IPBES 2019, pp.2–3).

GEO-6 chronicles a terrible record of environmental damage from biodiversity loss (including pollinators, coral reefs and mangroves), climate change and other air pollution, water pollution, ocean pollution and depletion, and land use change. As with ecosystem goods and services, these costs are difficult to express comprehensively in monetary or other terms, but GEO-6 confirms the level of many of the costs involved cited earlier (page numbers in what follows refer to GEO-6): For example, exposure to indoor/outdoor air and water pollution costs at least 9 million lives annually (p.78). Millions more suffer from ill-health and loss of livelihoods. Pollution-related costs have been estimated at USD 4.6 trillion annually (p.9). 29% of global land area is classed as a ‘land degradation hotspot’, affecting 3.2 billion people (p.203) and costing USD 6.3–10.6 trillion (p.374).

While no individual weather event can be attributed to a warming climate, the frequency and severity of extreme events are increasing due to a warming climate. As Lomborg (2020) notes, even similar events to those in the past are now more damaging because of both economic and population growth. Between 2010 and 2016, an average of around 700 extreme events each year cost an average of USD 127 billion per annum (Watts et al. 2017, pp. 615–616). While 90% of the losses came from high and upper-middle income countries, the less than 1% of the losses from low-income countries amounted to around 1.5% of their GDP, a much higher proportion than in high-income countries, and was almost all uninsured. Gupta and Ekins in UNEP 2019 (p.xxix) cite estimates of water-related health costs of about USD140 billion in lost earnings and USD 56 billion in health costs annually. From 1995 to 2014, 700,000 people died and 1.7 billion people were affected by extreme weather events costing USD 1.4 trillion (UNEP 2019, Fig. 4.1, p.80). As with biodiversity loss, the committed climate change that exacerbates these events is irreversible. They will continue to ravage human communities, perhaps with increased intensity, for the foreseeable future.

There is no doubt the scale of the ‘triple-de’ challenge is ambitious. Carbon-based fossil fuels have powered most of the world’s economic activity for more than two hundred years, since the use of coal to fire steam engines induced the Industrial Revolution. Oil, gas and coal currently make up almost 80% of primary energy use. [World Energy Outlook Special Report 2017: Energy and Climate Change, International Energy Agency] It therefore seems reasonable to ask what will be the cost of decarbonisation, detoxification, dematerialisation and repurposing infrastructure and behaviours to deliver a sustainable economy?

## The costs of environmental protection

The focus of this section is the costs associated with the task of tackling climate change and promoting sustainability noting that all environmental concerns are exacerbated by climate risks. We begin by noting that it is the stock of greenhouse gases that causes global warming, not the annual emissions. This means that keeping the global temperature at any level means transitioning to a net zero emissions world, because it is by definition the only way to stabilise the stock so the temperature will stop rising. This means humanity either manages the transition to temperature stabilisation or nature does it for us by depopulating and deindustrialising the planet.

If humanity chooses swift decarbonisation in line with the target of the Paris Agreement, many of the behaviours, technological networks and institutions of the last century look set to be devalued or stranded. Economies which are disproportionately dependent on fossil fuels in their production and trade may have higher transition costs than others, particularly where they lack flexible and responsive institutions (this is a problem for some gulf states, but even for more diversified but still ‘carbon entangled’ states like Russia, Nigeria, Kazakhstan, Indonesia or Poland). In other cases, such as coal in India or oil in Venezuela, fossil fuel-based organisations provide a significant part of the formal or informal welfare state of a nation or region. Such fossil fuel–dependent countries represent almost one-third of the world’s population (Peszko et al. 2020). Many have weak and inflexible institutions, limited access to global finance and pressing challenges of poverty, conflict and violence.

Having accepted the imperative of a low-carbon transition, it is necessary to acknowledge, up-front, that large-scale change will mean winners and losers and the losers will suffer dislocation. A quick scan of the global political economy tells us that these concerns need to be carefully managed. Because they can lead to delay, backlash, resistance and resource wastage. Indeed, they can make it hard for any economy to adjust to the forces of technological and structural change. Understanding the political economy is therefore necessary to manage change and enable all participants to profit from improved economic and social conditions. However, these concerns should not be falsely conflated with economic concerns regarding the true potential of environmental policies if implemented. It is the task of policymakers to limit the perceived reasons to slow or block necessary change.

Ensuring a just transition will be crucial for maintaining social cohesion and economic justice and enabling the climate transition to unfold. This requires enabling institutions that reskill, retool and compensate affected workers. It also requires policy responses to compensate consumers who face disproportionately higher costs (for example through temporary increases in energy or transport bills) and policy support for people living in towns and peripheral regions away from more dynamic urban centres better placed to manage change.

Economic theory and history also suggest that economies that embrace change, with diversified assets and flexible institutions are better able to manage structural adjustment (Zenghelis et al. 2018). Such economies do not inhibit the flow of resources from declining, low-productivity sectors to new, more productive sectors. They encourage, manage and steer it to gain competitive advantage (see Combes and Zenghelis 2014).

This section builds on this historical evidence and argues that addressing our resource and environmental challenges necessitates rapid innovation in technologies, behaviours and institutions. Section “Innovation, endogeneity and path-dependency” explains the dynamics of innovation and the opportunities to profit deliberately from its path-dependent nature.

As we find better ways of consuming, producing and living, we are likely to see complementary changes in behaviour, institutions and social norms (Section “Induced innovation and learning-by-doing”). In section “Networks, spillovers and contagion”, we will highlight the numerous immediate co-benefits associated with a transition. These, together with incentives from new policy drivers, are key to pushing early voluntary action on decarbonisation and resource efficiency based on near-term self-interest. For example, sprawling, congested, polluted cities with inefficient infrastructure and outmoded energy technologies do not in general attract highly skilled labour and act as a drag on GDP growth and wellbeing. This challenges the fallacy that a transition to a carbon–neutral economy is bound to make us worse off before it makes us better off. As expectations overcome inertia, tipping points can lead to rapid network shifts in key technologies and behaviours (Section “Feedbacks in preferences, behaviour and expectations”). This is not the context of standard growth models (Section “Innovation, endogeneity and path-dependency”) which struggle to capture structural change and integrate dynamic, increasing returns. Such models have a structural bias that tends to systematically overestimate costs of transition.

Overstating costs undermines the case for early action (Section “What does this mean for ‘green growth’?”). But it also delays investment and innovation necessary to facilitate a cost-effective transition. When change does come, governments and businesses caught unprepared risk being saddled with stranded assets and uncompetitive, outmoded forms of production. We conclude by showing that the virtuous dynamics associated with an accelerated and productive transition are unlikely to materialise without leadership to steer investment and innovation (Section “Ways forward”).

### Innovation, endogeneity and path-dependency

Innovation is essential in determining our ability to decouple growth and consumption from environmental degradation and resource use. Several climate economic models have attempted to incorporate innovation (see, for example, Popp 2004 and Bosetti et al. 2006). However, these models usually miss out important firm-level and sector-specific processes, spillovers and interactions or the role for mission-orientated, targeted R&D efforts.

Innovation does not just ‘happen’. It relies on path dependencies of three kinds: (1) research and development, (2) deployment and uptake, (3) network effects and economies of scale (Aghion et al. 2014). Strong inertia and high switching costs make it initially difficult to shift the innovation system from dirty to clean technologies without direct policy intervention. But once they reach a tipping point where expectations change rapidly and technologies switch from one network to another, these effects go the other way (Krugman 1991; Matsuyama 1991).

#### Induced innovation and learning-by-doing

Positive and reinforcing feedbacks derived from reduced technology cost accelerate further deployment and investment in supporting networks, infrastructure and institutions. Indeed, this arises specifically because of powerful network effects and high switching costs. Investments in enabling infrastructure spur technology tipping points through generating network externalities. For example, as electric vehicle infrastructure is rolled out, the incentives to conduct research and development on electric cars increase relative to combustion engine (or fuel cell) vehicles.

#### Networks, spillovers and contagion

Hidalgo et al. (2007) and Mealy and Teytelboym (2017) used network analysis to demonstrate that it is easier for countries to become competitive in new green products that require similar production capabilities and know-how to existing sectors. As a result, green transitions are highly path-dependent: countries which successfully invest early in green capabilities have greater success in diversifying into future green product markets. This reinforced the findings of Aghion et al. (2012) who provide empirical evidence that a firm’s choice whether to innovate clean or dirty is influenced by the practice of the countries where its researchers/inventors are located and that firms tend to direct innovation toward what they are already good at.

Braun et al. (2010) use OECD patent data to show that both wind and solar technologies create knowledge spillovers at the national level. Using data on 1 million patents and 3 million citations, Dechezlepretre et al. (2014) suggest that spillovers from low-carbon innovation are over 40 percent greater than from conventional technologies (in the energy production and transportation sectors). These effects, plus the cost savings as new networks and institutions are established, explain why Acemoglu et al. (2012) make a powerful theoretical case to suggest that policy to support clean innovation can be temporary, because once the “clean innovation machine” has been “switched on and is running,” it can be more innovative and productive than the conventional alternative, with a positive impact on GDP levels and growth.

These findings suggest governments should focus on areas where they have complementary advantage and prioritise early targeted R&D in these sectors. It also suggests that low-carbon investment can ‘crowd in’ productive investment and *generate* growth. Policy can thereby influence both the direction and pace of change (Fischer and Newell, 2008; Farmer and Lafond, 2016). Policy should aim to catalyse change in technology networks and behaviours while generating knowledge spillovers by focusing less on static market failures and more on dynamic ‘market creation’ and ‘market shaping’. This is likely to require that new policy frameworks and institutions be re-purposed strategically so as to steer the economy in a sustainable and resilient direction, for example through the creation of new low-carbon regulatory bodies and public investment banks with strong sustainability mandates.

#### Feedbacks in preferences, behaviour and expectations

A key source of path dependence in socioeconomic systems is the presence of ‘complementarities’ in expectation formation. This occurs when the payoff to the whole group from working together is greater than the sum of the individual payoffs. In particular, ‘strategic complementarities’ arise when agents make individual decisions that affect each other’s welfare and one agent’s greater productivity makes *all* the other agents more productive. Research and development externalities (Romer 1990) and learning spillovers (Arrow 1962) in low-carbon technologies have these features—as more scientists start thinking about clean energy, more ideas and innovations emerge that other scientists can use. But technology is not the only source of rapid change and innovation. Behavioural, institutional and social innovation can guide demand-side factors relating to consumer preferences (Boyd et al. 2015).

Social norms can be defined as the predominant behaviour within a society, supported by a shared understanding of acceptable actions and sustained through social interactions (Ostrom 2000). Social feedbacks help make norms self-reinforcing and therefore stable. Formal institutions struggle to enforce collectively desirable outcomes without popular support. Acceptable standards of behaviour and social norms are the sources of law and ultimate drivers of legislative change (Posner 1997).

Regulations, taxes, subsidies or infrastructure investment such as cycle lanes or dense housing and public transport can aid the process of shifting norms. Build cycle lanes and people will buy bicycles. A potentially powerful role for policy is to provide reasons for people to change their expectations and behaviours (Young 2015). Social psychologists have long understood that solving coordination problems requires building expectations into models and generating ‘common knowledge.’ (Thomas et al. 2014). In low and middle-income countries, this can take the form of political processes that confer authority and capacity on public institutions not only to diffuse new innovations but also to generate agglomeration hubs (Collier 2017). This can help implement the legal and fiscal frameworks, as well as skills and infrastructure, to attract international knowledge through multi-national inward investment, trade and foreign finance. Institutional development is central to enable technological innovation, adoption and growth (Easterly and Levine 2003). It also allows domestic innovation to be diffused through universities, research institutes and high value-added urban employment. Far from acting as a constraint, decarbonisation may afford low-income countries an opportunity to accelerate growth by breaking new ground without relying on incremental change to legacy infrastructure which they lack. Analogous to the spread of mobile telephony, developing countries can leapfrog developed economies and increase access to basic electricity services by bypassing expensive and inefficient centralised electricity grid infrastructure and investing instead in distributed energy platforms (Alstone et al. 2015; Levin and Thomas 2016).

In all countries of all income levels, local technology clusters create positive spillover effects of lowering information, transaction and installation costs (Porter 2000). One study showed how social networks and dwelling proximity explained the clustering of photovoltaic panel installation by homeowners (Rogers 2010). Changing social norms can also add to the costs of polluting by putting pressure on legislators, as well as influencing strategic decision-making contexts, such as in the financial sector.

Investment norms can also shift as investors begin to perceive risk in high-carbon resource-intensive sectors previously deemed safe, for fear that assets (resources, physical infrastructure, human capital and intangible know-how) might become devalued or stranded in coming decades. Meanwhile, the risk premium attached to investment in clean sectors, previously considered exotic, falls as these are seen as more resilient to a low-carbon resource-constrained future (Bradshaw 2015; Zenghelis 2016). Pension funds and insurance companies are correspondingly cutting support for coal projects (Financial Times 2018). Meanwhile clean sectors outperform their peers in terms of financial returns (Friede et al. 2015; Clark et al. 2015). Governments should work to push firms and public institutions to better integrate climate risk assessment into investment decisions, through mandated disclosure standards and scenarios for stress-testing resilience to rapid future decarbonisation.

The point here is that actual or expected changes in policy, technology and physical risks—as well as the threat of litigation for loss and damage from climate change—could prompt a rapid reassessment of the value of a large range of assets as changing costs and opportunities become apparent (Stern and Zenghelis 2016). For many low- and middle-income countries, failure to act early could mean: markets are closed off to their products because they do not meet new standards and regulations in export markets, or they face border tax adjustments; higher costs of capital as multilateral and other investment banks withdraw from carbon-intensive sectors, and competitiveness declines as a result of being saddled with less productive or redundant legacy technologies. This raises the cost of ‘pollute now clean up later’ strategies and enhances the risk of early yet avoidable financial loss and the locking in to stranded assets, while accelerating the cost-effective transition to a clean economy.

#### Co-benefits and opportunities as a trigger

Changing expectations hold the key to overcoming inertia and unblocking a waiting game. This is underpinned by a growing appreciation of additional opportunities associated with a low-carbon transition (Hale 2018). These include not only commercial opportunities associated with deploying (and fabricating and exporting) cheap and increasingly competitive new clean technologies, but also benefits from reductions in waste and inefficiency, improved energy security and reduced particulate pollution and congestion from clean compact cities.

Not only are pollution externalities not priced, but environmentally degrading activities continue to be actively encouraged by policy. IEA estimates that in 2015 subsidies to fossil fuels were twice as large as those to renewables (van Asselt and Kulovesi 2017). Coady et al. (2015) estimate that eliminating subsidies for fossil fuels would have reduced global carbon emissions in 2013 by 21% while boosting net public revenues by 4% and reducing deaths from local air pollution by 55%.

The Global Commission on the Economy and Climate (2014) found that more than half and as much as 90% of the global emissions reductions required to meet an ambitious climate target could generate net benefits to the economy. These include health benefits from reductions in urban pollution, falls in traffic congestion, increases in efficiency or improvements in energy security and supply. Hallegatte et al. (2012) argue that compared with business-as-usual, green growth would mean immediate positive effects on the economy, such as co-benefits (e.g. reduced local pollution), growth in new ‘green’ sectors, and less energy price volatility via reduced dependence on fossil fuel imports. Higher income generates resources for investment in environmental quality and poverty eradication (Hepburn and Bowen 2013).

#### Complementarities, cascades and tipping points

Changes in expectations can overcome the burdens of history to become self-fulfilling. As enough players shift investment to deploy new technologies, learning and experience will push their price down, making further investment increasingly attractive relative to conventional technologies, where the gains from additional learning or scaling are smaller.

Very quickly, an economy can switch from one technology network to another as a newcomer becomes more attractive than the incumbent, until incumbent technologies, products and networks become obsolete. (Otto and Donges 2019).

**Fig. 3:**
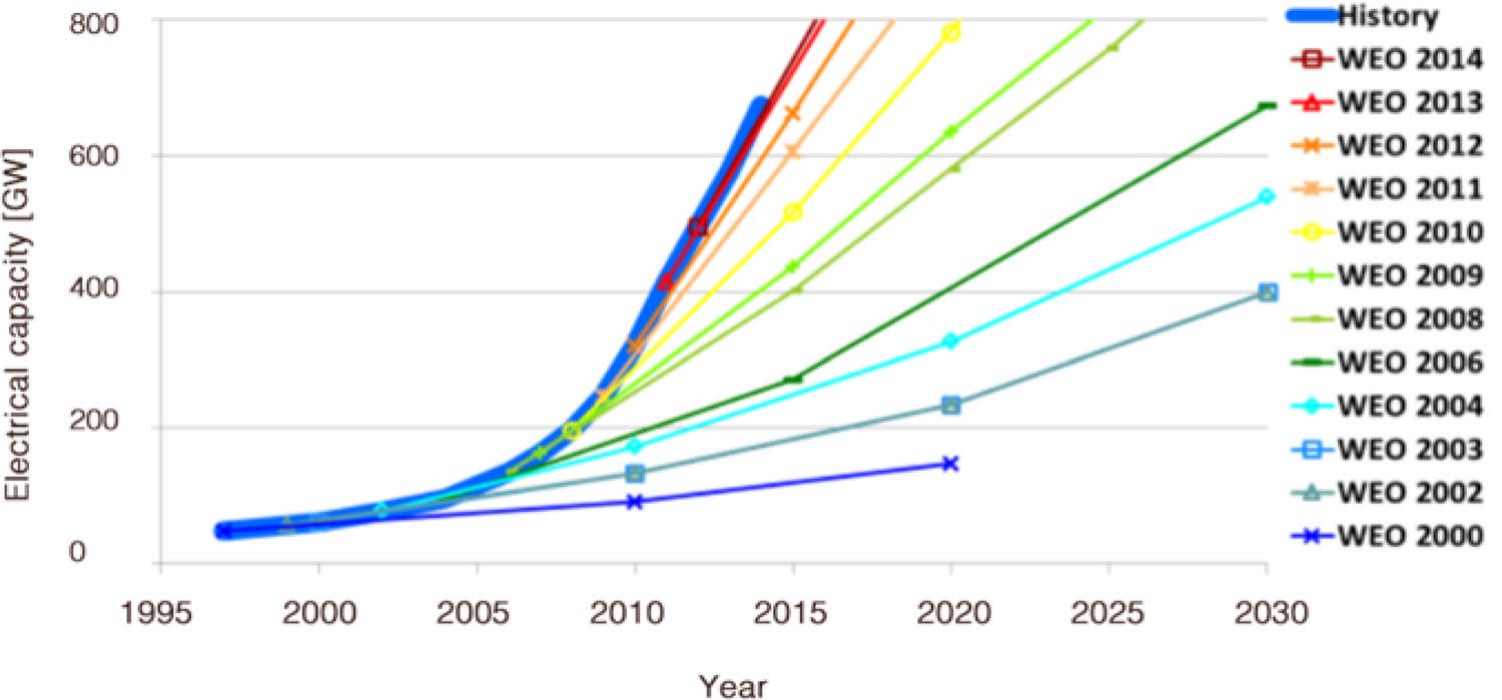
IEA renewable capacity forecasts, ex-hydropower; Source: Metayer, Breyer and Fell, 2015 (https://www.lut.fi/documents/10633/70751/The-projections-for-the-future-and-quality-in-the-past-of-the-World-Energy-Outlook-for-solar-PV-and-other-renewable-energy-technologies-EWG-WEO-Study-2015.pdf)

Failure to model the dynamics and positive feedbacks associated with structural change, not only in terms of economies of scale from production and discovery, but also the complementarities and feedbacks associated with contagion and systems tips, are the reasons why most economists have been caught out by the rapid nature of the early phases of the post-carbon transition. Figure 3 shows forecasts made by the International Energy Agency (IEA) for the deployment of renewable technologies compared with actual outturns. The IEA is arguably the leading authority on energy technologies, yet they systematically and repeatedly underestimate the deployment of renewables and correspondingly overestimate the costs.

**Fig. 4:**
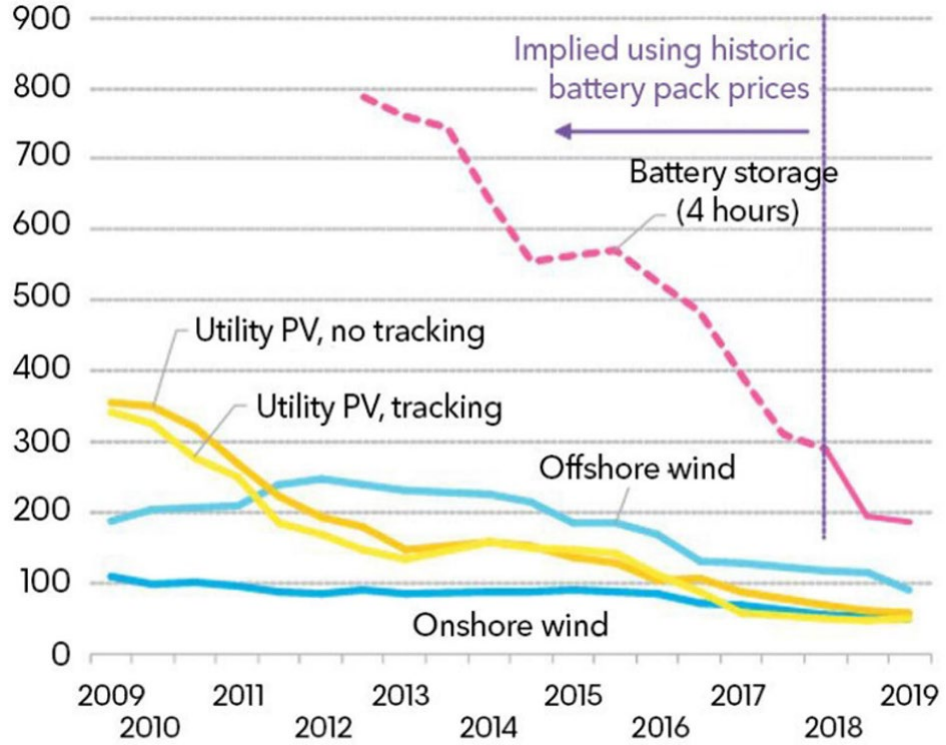
Renewables, levelized cost $/MWh, 2018 real. Source Bloomberg NEF: country weighted average using latest capacity additions. Storage based on utility-scale Li-ion battery running at a daily cycle and includes charging costs assumed to be 60% of wholesale power price in each country

The IEA are not alone. Few economists predicted the precipitous fall in the price of renewable technologies. Solar photovoltaic (PV) costs fell 44 per cent^2^ in the two years to the end of August 2017 and have fallen by 83 per cent since 2010,^3^ a period over which the price of wind turbines has dropped 35 per cent.^4^ They have already become the cheapest source of energy in many world regions (Fig. 4). And the rate of change shows no sign of slowing. Regardless of the impact on emissions, the world now faces the prospect of cheaper energy and transport costs than would otherwise have been the case. The market alone would not have delivered this and no models predicted it. These opportunities can crowd-in resources to pay for more expensive but necessary technologies, such as large-scale direct air capture technologies.

A decade ago, to the authors’ knowledge, no one predicted that renewables would become the dominant source of energy investment by the second decade of this century, surpassing coal, oil, gas, nuclear and hydro combined.^5^ Few predicted the expansion in LED lighting from less than 5 per cent to more than 40 per cent of the global market in the past 6 years.^6^ Yet the processes underlying these developments are predictable, and may be expected to apply to some of today’s frontier technologies such as green hydrogen.

All this suggests that structural shifts, when they happen, can progress surprisingly fast following a long period of apparent inaction and inertia. This further highlights the importance to policymakers of explicitly integrating co-benefits, uncertainties, path dependencies and irreversible thresholds into their comprehensive project assessments, recognising the limitations of standard cost–benefit analysis.^7^

### The limitations of standard economic models

The prevalence of reinforcing feedbacks and tipping dynamics largely invalidates traditional analytical approaches based on assumed patterns of incremental change taking the world as given as embodied in IAMs. As a result, not only are models’ power to inform policy limited, for example by missing the effect of credibility on the formation of expectations, but also the expected costs of decarbonisation will likely be overstated.

Most conventional approaches to determining the efficient path for coping with climate change, including standard Integrated Assessment Models (IAMs), need to presuppose the technologies, tastes, preferences and behaviours that will dominate in the decades and centuries ahead to give the model structure (Zenghelis 2018a, b). But these imposed structural assumptions are precisely the things we want to know when predicting the costs of transitioning to low-carbon networks, processes and behaviours. Marginal static analysis techniques, which assume the wider world is unchanged by the intervention, are inappropriate for non-marginal, structural changes of global proportions.

Consider, for instance, the neoclassical DICE and RICE models^8^ of Nordhaus. These are among the models most widely used to quantify the costs and benefits of climate policy. In this framework, capital and labour are used to produce a single consumption good. The total productivity of these factors depends upon a single technology parameter, which is imposed and grows exogenously over time. Emission intensity of production also grows exogenously. This leaves little of real interest that the model can tell us.

That said, simplified models like DICE can be useful for transparent sensitivity analysis of key parameters. Grubb and Wieners (2020), for example, challenge the assumption of temporal independence whereby abatement costs in one period are assumed unaffected by prior abatement. They use DICE to illustrate how a ‘slow carbon price ramp’ approach is inefficient in the case where carbon abatement costs are shaped by innovation. They use the model to illustrate how once a technology becomes sufficiently competitive, it starts to change the entire environment in which it operates. In such cases, the optimal strategy involves much higher initial investment in abatement. For the same reasons, rather than working along an abatement cost schedule picking off the cheapest options first, it might make better sense to start with some of the most expensive technological options to bring their costs down faster (Vogt-Schilb et al. 2018).

More complex Computable General Equilibrium (CGE) models, commonly used in IAMs, are no better equipped to handle multiple equilibria and the transition from one equilibrium to another (Mitra-Kahn 2008). Dynamic CGE models rely on ‘backward-looking’ adaptive expectation formation or ‘forward-looking’ rational expectation formation to generate a smooth, efficient, balanced equilibrium pathway. Both fail to account for the importance of expectations in driving equilibrium shifts.

Structural macroeconomic models lack the restrictive ‘micro-foundations’ and optimisation assumptions of CGE models, but they are reliant on estimated historical time series to inform behavioural parameters. This also renders them inadequate in making *ex ante* predictions of future structural change which, by definition, will look very different from the past.

**Fig. 5:**
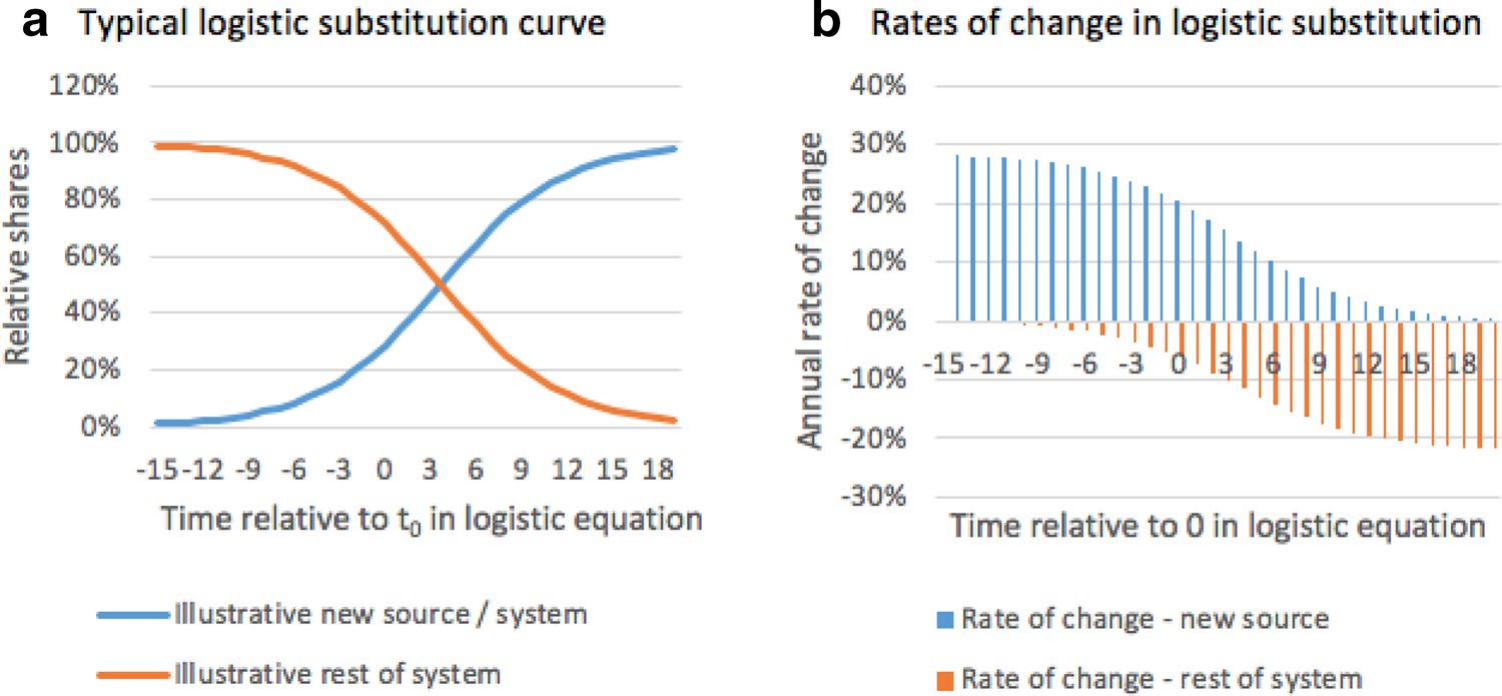
Systemic network transitions Source: Grubb 2018

Grubb (2018) points out that if technology substitution in a structural transformation is intrinsically dynamic and irreversible, with technologies “striving” for dominance, new technological systems can rapidly scale-up and displace older ones in a way that cannot be determined from examining past data in that sector (Fig. 5). In such cases, technological penetration is best described in the form of a logistical substitution or S-curve:

$${\text{deployment}}\;{\text{at}}\;{\text{time(t)}} = \frac{{{\text{MAX}}}}{{1 + ae^{ - k(t - t0)} }}.$$

Models applying ‘historical futures’ analysis by way of incremental improvements in energy and carbon efficiency, or which focus exclusively on what policies have worked in the past, are often doomed to under-predict the scope and pace of change simply because when it comes to structural transitions, the past is no guide to the future.

Incorporating features of path-dependent phenomena—switching costs, inertia, knowledge spillovers, network effects, feedbacks, and complementarities—into economic models leads to a multiplicity of ‘equilibria’, each dependent on a different development path (Aghion et al. 2014).

This makes it very hard to predict costs and benefits over the next few decades, and explains why conventional economic models, even though they often make unrealistic assumptions about optimal policies (such as the application of a uniform global carbon price) which ought to understate the costs of decarbonisation, in general systematically overstate the costs of decarbonisation. It also means that the answer to the net cost of decarbonisation question is endogenous. Innovation in technologies, behaviours and institutions is shaped by action which will determine costs and benefits. Traditional economic models have their place, but their limitations need to be understood.

### What does this mean for ‘green growth’?

Spurious model projections have spawned often diametrically opposed and mostly flawed assessments of our ability to live sustainably. One set of authors uses the high-cost projections to question whether ambitious mitigation offers value for money. Integrated assessment models like RICE, DICE and FUND conclude global carbon prices should start low and follow a ‘slow policy ramp’. In his Nobel Prize speech, William Nordhaus, the architect of RICE/DICE, described global temperatures of 3 or 4 degrees above preindustrial levels—levels climate scientists view as potentially catastrophic—as ‘optimal’ given the high costs of adjustment in his models. This is a view most recently endorsed by Lomborg (2020).

A second camp draws the reverse conclusion. They argue that if absolute decoupling between consumption and emissions is prohibitively expensive, then the only feasible approach to decarbonisation and living within planetary limits is reduced consumption and output—often termed ‘degrowth’ (Jackson 2016; Hickel and Kallis 2019).

Prosperity and wellbeing is about more than just GDP growth. But it is important not to mistake output growth with growth in material inputs such as fuels, minerals, ecosystem services and capital equipment. This ignores the dynamic scale economies associated with innovation. Unlike material resources, knowledge is weightless and when used is hard to deplete **(**Quah 1999). Indeed, knowledge builds on knowledge: one of the sources of endogenous growth is that constant or increasing returns to ideas can overcome diminishing returns to physical capital (Weitzman 1996; Hepburn and Bowen 2013).

Innovation can reduce material throughput for each unit of GDP value created and will do so more as electricity generation is decarbonised. This is reflected in the increasing importance in national income of intangible, knowledge-products—computer software, new media, electronic databases and libraries, and online services. Endogenous growth theory developed by Romer (1990) highlighted how increasing returns to ideas overcome the diminishing returns to factors like labour and capital generating resources for further investment.

Degrowth is not the place to start when there are so many untapped opportunities associated with sustainability, especially as history suggests that declining economies are neither efficient in their use of resources nor clean. In any case, economic contraction would be among the most expensive solutions, significantly undermining welfare (Zenghelis 2019).

From a practical perspective, degrowth is likely to prove a hard sell, particularly in parts of the rapidly developing, populous world where growth is (rightly) seen as a primary means to eradicate poverty. In any case arguments for ‘degrowth’ overlook the extent to which the loss of natural capital is already constraining economic growth, in line with the perception that natural capital, rather than manufactured capital, is now the scarce production factor (UNEP 2011).

The approaches adopted by both the degrowth and wait-for-technology camps serve to delay action, using models which overstate the costs of decarbonisation and discourage businesses and policymakers from investing in new technologies and infrastructure. The problem is worse than one of spurious precision and bad forecasts. Those who use static models not only *get* the future wrong, they *make* the future wrong by generating what game theorists call an inferior Nash equilibrium.^9^ To the extent that such models are believed, they become self-fulfilling, reflecting once again the key role of expectations.

Paul Romer, a key architect of the endogenous growth framework, focused on the dynamics of growth embodied in innovation, network effects and complementary feedbacks. He emphasised path-dependency and the power of decisions today to profoundly shape and reshape the future. Romer states:

“What the theory of endogenous technological progress supports is conditional optimism, not complacent optimism. Instead of suggesting that we can relax because policy choices don’t matter, it suggests to the contrary that policy choices are even more important than traditional theory suggests.”

Models that fail to understand this will consistently get (and make) the future wrong. Our ability to cost-effectively transition to a sustainable economy will be a function of the decisions we take today.

### Ways forward

All this suggests that the economic toolkit can be put to better use helping policymakers understand and steer path-dependent processes. Economics will need to draw from a range of disciplines, integrating perspectives from the social, physical, and natural sciences as well as the humanities. This needs to include history, spatial geography, planning and social psychology, game theory, anthropology, epidemiology, computer and network science and sociology to derive a richer and more valuable understanding with which to guide decision-makers (Haldane and Turrell 2018).

One approach suggests that, in addition to theoretical and network-based analysis, dynamic models of the economy should be coupled with models of opinion dynamics and behaviour by use of agent-based models. These explicitly reflect interactions between heterogeneous, networked individuals in place of conventional ‘representative agents’ (Farmer and Foley 2009). As a result, they offer insights into the probability and processes through which economies shift from one equilibrium to another (Mealy and Hepburn 2019).

A number of authors now recognise that better understanding the processes and innovations which generate the cascades of tipping described above is more valuable to policymakers than speculative projections of costs. By taking advantage of the inherent domino effect of rapid, self-amplified and contagious change, policymakers can leverage highly sensitive “tipping interventions” that deliver outsized impact (Schellnhuber et al. 2016; Farmer et al. 2019) which could hasten global decarbonisation (Tàbara et al. 2018)^10^. Rather than focus on predictions based on ‘historical futures’, information on innovation processes can be gleaned by looking at historical transitions, such as the change from kerosene use to electricity, horse and cart to combustion engines and photographic film and records to digital photos and music (Zenghelis et al. 2018).

The trigger could be, for example, a specific climate or energy policy or a breakthrough technology (such as cheap and effective energy storage). The point is that policies, institutions, and technologies reinforce each other in a positive feedback loop precipitating take-off and diffusion of sustainable forms of production. Targeted tipping interventions could simultaneously precipitate mutual reinforcement and overcome barriers to decarbonisation (Rickards et al. 2014). Our analysis of strategic complementarities in section “Feedbacks in preferences, behaviour and expectations” showed how agents base their decisions on how they anticipate others will act (van der Meijden and Smulders 2017). This collectively underscores the importance of leadership and clear credible policies to guide investors (Aral 2011) and kickstart the green innovation machine through public investment, guarantees and risk sharing.

The low-carbon transition is likely to have influence beyond rich-world economies, with many developing countries encountering significant structural shifts of their own. Rapid change in technologies, policy frameworks and markets are rendering traditional industrial development routes less viable. Development agencies and multilateral financiers are seeking to diversify assets and lock into profoundly different sustainable pathways (Peszko et al. 2020). Multilateral funding supported by enhanced technological diffusion can promote both development and decarbonisation and allow countries to leverage global finance to deploy green technologies and generate capacity in new technologies (Hidalgo et al. 2007; ECA 2016).

None of this is to say that a *managed* low-carbon transition is inevitable. Political economy barriers and effective lobbying by incumbents could continue to slow progress in hard-to-transition sectors such as industry (in particular metals, ceramics, chemicals cement and plastics), aviation, shipping and haulage (Energy Transition Commission 2018). This could prevent a shift to net zero in time to avoid critical climate risks. But, in line with Paul Romer’s conditional optimism, the fact that we may not profitably do what is necessary in time does not mean we cannot do so. A prerequisite for doing so would be leadership and a common understanding of the costs and opportunities, a role facilitated by the appropriate application of the economists’ toolkit.

On a final note, shortly after the publication of the GEO-6 report, and after this paper was first drafted, the world was transformed by the Covid-19 pandemic. A number of studies have highlighted the fact that the move from pandemic lockdown and rescue to post-pandemic recovery offers an opportunity to ‘build back better’ so as to secure resilient, inclusive and sustainable growth (Hepburn et al. 2020). There is a growing realisation that the growth model that followed the great financial crash marked a wasted opportunity (Stern et al. 2020).

The post-Covid recovery marks an opportunity to invest surplus desired saving into a broad range of productive complementary assets, including physical, human, knowledge, social and natural capital, to secure future prosperity (Agarwala et al. 2020). Because these investments in society’s comprehensive wealth utilise a surplus of desired global savings, which has pushed global real risk-free interest rates below zero, the benefits of public investment based on mounting public debt are likely to exceed the costs, for most countries able to borrow in their own currency (Zenghelis et al. 2020). As part of a coordinated and strategic policy framework, they also have the potential to leverage in far larger sums of private investment (Green Finance Taskforce 2018). Low- and middle-income countries have less scope to rely on public borrowing and are likely to need augmented support from high-income countries to access finance and technologies necessary to secure sustainable growth, once the pandemic has been brought fully under control.

## Conclusions

Given limited time and resources available to address mounting concerns, traditional economic approaches to assessing our response to environmental challenges are not only flawed, they are dangerous. Physical science shows the threat posed by complex adaptive systems surpassing critical thresholds and irreversible tipping points.

A key conclusion of this analysis is that starting early by credibly steering expectations, inducing innovation and directing investment is in all cases better than delay. Actively managing a transition to a low-carbon, sustainable economy means strong policy signals which allow governments and businesses to avoid investing in high-carbon, resource-intensive infrastructure, technologies and assets that are liable to become stranded, devalued or redundant before the end of their working lives. For all these reasons, we conclude that ‘grow now, clean up later’ is the second highest cost option (assuming ‘clean up’ is possible). Only the existential costs of never cleaning up are higher.

This paper has shown why delay is triply bad:

1. Climate damages mount as the stock of greenhouse gases go up, and challenges from depleted and degraded resources mount, especially when such degradation, such as in respect of land and marine ecosystems, is irreversible.
2. Climate and resource depletion damages mount much more quickly as productivity growth is eroded through endogenous effects of devalued and destroyed capital (Dietz and Stern 2015)
3. Lock-in of resource- and carbon-intensive infrastructure, behaviour and institutions and reduced innovation in substitutes increases the cost of attaining sustainable pathways.

Early action can induce creativity and innovation and generate tipping points as feedbacks and dynamics become reinforcing. This is why the correct answer to the question ‘what will it cost to decarbonise in the long run?’ is ‘it is endogenous’. It depends on the choices and actions we take today and in the future. A common understanding that a managed low-carbon transition is both imperative and affordable is the most effective way to induce a rapid transition at least cost.

But understanding the endogeneity of the system and the role of expectations in determining the evolution of costs and competitive advantage suggests the whole notion (and associated industry) of forecasting the cost of decarbonisation is somewhat misplaced. The economic toolkit needs to be put to more creative use to help policymakers understand and steer path-dependent processes.

This paper concludes that analytical insights can potentially allow policymakers to leverage highly sensitive “tipping interventions”, by taking advantage of the inherent domino effect of self-amplified and contagious dynamics. We are already seeing increasing returns to scale in discovery and production, and we are seeing very powerful complementarities and positive feedbacks in systems. Transformative change is gripping key global energy and transport sectors after decades of inertia: new networks, behaviours and institutions are replacing old. Policymakers and businesses are increasingly adopting risk management and hedging strategies that limit investment in conventional technologies and behaviours that may be rendered stranded or devalued. This is not the context of standard growth models, yet policy is needed to support and accelerate this momentum.

Recent evidence suggests the short-term GDP impacts of well-designed environmental action could be positive, crowding-in rather than ‘crowding out’ the drivers of future growth. Moreover, much environmental harm is irreversible, most obviously biodiversity loss and tipping points associated with a changing climate. This paper provides evidence that not only makes the environmental case for action, in terms of its benefits for human health and welfare, it also shows how such action can generate economic returns in terms of productivity, jobs and income and reduce the costs of meeting any emissions and resource use targets. A cost-effective low-carbon, resource-efficient transition can generate a cleaner, quieter, more secure, innovative, and productive economy for all countries at all stages of development.

But our optimism is conditional. It requires credible and ambitious action in the near term to avoid catastrophic and irreversible environmental risk by overcoming continued inertia in unsustainable activities, and time is not on our side. There is no room for fatalism and complacency. Using the wrong economic tools to assess the costs of systemic technological and behavioural transformation to address climate change delays action. Understanding the dynamic process of innovation, on the other hand, means putting investment, innovation and technical change at centre stage.

## Footnotes

1 https://edition.cnn.com/2019/01/02/health/plane-crash-deaths-intl/index.html.

2 https://about.bnef.com/new-energy-outlook/.

3 https://www.linkedin.com/pulse/scenarios-solar-singularity-michael-liebreich/.

4 https://about.bnef.com/new-energy-outlook/.

5 See: https://www.bloomberg.com/news/articles/2019-09-05/clean-energy-investment-is-set-to-hit-2-6-trillion-this-decade.

6 https://www.strategyr.com/MarketResearch/market-report-infographic-chip-on-board-light-emitting-diodes-cob-leds-forecasts-global-industry-analysts-inc.asp.

7 Standard social cost–benefit analysis is often limited to a narrow market failure framework, omitting broader spill overs and dynamic interactions. The UK Green Book, used by officials to assess public projects, for example, states that: *‘5.5 Social CBA and Social CEA are “marginal analysis” techniques. They are generally most appropriate where the broader environment (e.g. the price of goods and services in the economy) can be assumed to be unchanged by the intervention. These techniques work less well where there are potential non-marginal effects or changes in underlying relationships. This is due to the difficulties inherent in pricing such changes. It is possible to adapt Social CBA in these cases, for example when appraising the cumulative impact of interventions on Natural Capital. Significant non-marginal issues which cannot be reflected in Social CBA need to be appraised and considered at the long-list stage.’ – The Green Book.*

8 https://sites.google.com/site/williamdnordhaus/dice-rice

9 https://www.bennettinstitute.cam.ac.uk/blog/mind-over-matter-how-expectations-generate-wealth/.

10 See Oxford Martin School, “Programmes: Post-carbon Transition,” www.oxfordmartin.ox.ac.uk/research/programmes/post-carbon.
